# Molecular Characteristics of *Brucella* Isolates Collected From Humans in Hainan Province, China

**DOI:** 10.3389/fmicb.2020.00452

**Published:** 2020-03-27

**Authors:** Zhenjun Li, Xu-ming Wang, Xiong Zhu, Miao Wang, Hai Cheng, Dan Li, Zhi Guo Liu

**Affiliations:** ^1^State Key Laboratory for Infectious Disease Prevention and Control, National Institute for Communicable Disease Control and Prevention, Chinese Center for Disease Control and Prevention, Beijing, China; ^2^Hainan Provincial People’s Hospital, Haikou, China; ^3^Sanya People’s Hospital, Sanya, China; ^4^Ulanqab Centre for Endemic Disease Prevention and Control, Jining, China; ^5^Inner Mongolia Autonomous Region Center for Comprehensive Disease Control and Prevention, Huhhot, China

**Keywords:** *Brucella melitensis*, *Brucella suis*, multiple loci variable number tandem repeats analysis, single-nucleotide polymorphism, Hainan

## Abstract

Brucellosis has been reported in several regions of Hainan Province, but the extent of the disease has not been fully elucidated. Conventional biotyping methods, multiple locus variable number tandem repeats analysis (MLVA), and single-nucleotide polymorphisms (SNPs) from draft genome sequencing were employed to characterize the strains. There were four biovars (*Brucella melitensis* bv. 1, 2, and 3 and *Brucella suis* bv. 3) detected, which showed that the biovar diversity of *Brucella* in Hainan is higher than in other areas of China. Both *B. melitensis* bv. 3 and *B. suis* bv. 3 were dominant species and showed epidemiology patterns that were compatible with both southern and northern China. Eight of MLVA-11 genotypes were known (31, 111, 116, 120, 136, 291, 297, and 345), and the remaining seven were novel (HN11-1 to HN11-7); these data showed that *Brucella* strains in this study had multiple geographic origins and exhibited characteristics of origin and evolution of co-existing imported and Hainan specific lineage. A total of 41 strains were found, belonging to 37 unique genotypes that each represented a single strain, which suggests that these strains were not directly related epidemiologically and indicates that the epidemic characteristics of human brucellosis in Hainan was dominated by sporadic strains. The high HGDI values were observed in MLVA-8, MLVA-11, and MLVA-16 among two species, suggesting considerable genetic diversity among these species. MST is characterized based on MLVA-16 that was found both throughout China and on a global level and showed that strains of this study had significant genetic differences with strains from many parts of the globe and seemingly represent a unique genetic lineage. Whole-genome SNP analysis showed that four *B. melitensis* were closely related to strains from China’s northern provinces, and the source of infection was partly of human brucellosis in this province that may have been from these regions. The *B. suis* were closely related to strains from the United States, and further investigation of the transportation of animals, such as pigs, is needed to elucidate the origins of these strains.

## Introduction

Globally, brucellosis is a zoonosis affecting a wide range of domestic and wild animals and humans (Marvi et al., 2018). Due to its complex nature, brucellosis remains a serious threat to public health and livestock in developing countries (Khan and Zahoor, 2018). The genus *Brucella* has traditionally been divided into six species based on host preference, *B. abortus*, cattle; *B. melitensis*, sheep and goats; *B. suis*, pigs; *B. canis*, dogs; *B. ovis*, sheep; and *B. neotomae*, wood desert rats (De Figueiredo et al., 2015). Recent isolates from humans (*B. inopinata*), aquatic mammals (*B. pinnipedialis* and *B. ceti*), and a common vole (*B. microti*) have been recognized as new *Brucella* species (De Figueiredo et al., 2015). At present, human brucellosis cases had been reported in almost all China provinces since 2010 (Lai et al., 2017). In Hainan Province, which is located at the southernmost part of China, there were no historically reported brucellosis cases, and only one human brucellosis case was reported in Danzhou in 1985, brucellosis cases significantly increased in 2017 between humans (Wang et al., 2019), but the available information on the extent of the disease and relatedness among strains is unknown. Characterizing the circulating strains is a critical sector in understanding brucellosis in the epidemic area (Ledwaba et al., 2019). Accurate discrimination relatedness among strains with the MLVA assay is necessary to determine the source, origin, and geographical spread of infection (Whatmore et al., 2016). Moreover, the characterization of the genome of *Brucella* species with WGS provides excellent genetic resolution and resolve intraspecies relationships for closely related species (Wattam et al., 2014; Abou Zaki et al., 2017). Presently, whole-genome data from many species of *Brucella* spp. strains have been published and are available in NCBI (Delvecchio et al., 2002; Pisarenko et al., 2018). The purpose of this study is to analyze these *Brucella* strains using bio-typing and to arrange the molecular scheme including MLVA and WGS to elucidate prevalence characteristics and relatedness among strains for epidemiological purposes, and to provide useful information for the control and prevention of brucellosis in Hainan Province.

## Materials and Methods

### Ethics Statement

This research was conducted according to the principles of the Declaration of Helsinki. The study is a retrospective investigation of historical strain collections using molecular typing methods, and the study protocol was approved by the Ethics Committees of the National Institute for Communicable Disease Control and Prevention. Informed consent was obtained from all of the patients prior to diagnosis and all patient data were anonymized. *Brucella* spp. were isolated from patients’ blood samples and were used for diagnosis of disease and following the confirmation of their consent.

### Bacterial Strains

A total of 41 strains were examined in this study. These strains were collected from nine counties in Hainan Province from 2009 to 2019. A total of 41 strains were recovered from 41 patients: 39 of them were collected from the blood of patients, and the other two strains were recovered from cerebrospinal fluid and vertebral puncture fluid. The isolates and biovar of the strains were identified using standard procedures (Alton et al., 1975). All isolates were identified as *Brucella* species on the basis of morphology and conventional identification methods according to standard biotyping procedures, including the need for CO_2_ for growth, H_2_S production, sensitivity to thionin (10 and 20 μg/ml), basic fuchsin (20 μg/ml), and agglutination with mono-specific antiserum for A and M antigens and phage lysis test [Tbilisi (Tb); Berkeley (Bk2); Weybridge (Wb)] (Al Dahouk et al., 2003; OIE, 2018). Reagents and pages were obtained from the China Institute of Veterinary Drug Control, which is China’s National Reference Laboratory for Animal Brucellosis. *B. melitensis* 16M (BM), *B. abortus* 544 (BA), and *B. suis* 1330 (BS) reference strains were used as experimental controls. The DNA of the strains was extracted with a Nucleic Acid Automatic Extraction System (LLXBIO China, Ltd., China) using a single loop of fresh *Brucella* cells that were grown for 48 h according to the manufacturer’s instructions.

### *Brucella* MLVA-16 Genotyping Scheme

MLVA-16 was performed as previously described (Liu et al., 2017). The 16 primer pairs were divided into three groups: Panel 1 (MLVA-8: eight loci including bruce06, bruce08, bruce11, bruce12, bruce42, bruce43, bruce45, and bruce55), panel 2A (three loci including bruce18, bruce19, and bruce21), and panel 2B (five loci including bruce04, bruce07, bruce09, bruce16, and bruce30); MLVA-11 (panels 1 and 2A), and MLVA-16 (panels 1, 2A, and 2B). PCR amplifications were performed in 20-μl reaction volumes. A total of 5 μl of PCR products for the 16 loci were denatured and resolved by capillary electrophoresis on an ABI Prism 3130 automated fluorescent capillary DNA sequencer (Applied Biosystems). Fragments were sized after comparison to a ROX (carboxy-X-rhodamine)-labeled molecular ladder (MapMaker 1000; Bioventures, Inc., Murfreesboro, TN, United States) and Gene Mapper software version 4.0 (Applied Biosystems). The fragment sizes were subsequently converted to repeat unit numbers using a published allele numbering system (Scholz and Vergnaud, 2013).

### Analysis of the MLVA Data

BioNumerics version 7.6 software (Applied Maths, Belgium) was used to analyze the MLVA-16 assay data. Both the categorical coefficient and the unweighted pair group methods were applied to the clustering analysis. MLVA-11 was used to investigate the geographical origins between our isolates (De Massis et al., 2015; Liu et al., 2019) (Supplementary Table S1) and 528 isolates from the MLVA bank including the non-available group (2), the Africa group (4), the America group (75), the West Mediterranean group (77), the China group (97), and the East Mediterranean group (273) (Supplementary Table S2). Minimum-spanning trees were constructed using the goeBURST algorithm with PHYOVIZ 2.0 (Nascimento et al., 2016). The MLVA-16 approach was applied to the genetically related investigation between strains from those reported in China (*n* = 367) (Supplementary Table S3) and at the global level (*n* = 2687; 2124 in *B. melitensis* and 563 in *B. suis*) (Supplementary Tables S4_1, S4_2). The genetic diversity of *Brucella* strains in this study was calculated based on the Hunter–Gaston Diversity Index (HGDI) according to a previously published method (Hunter, 1990).

### Single-Nucleotide Polymorphism (SNP) Analyses Based on the Draft Genome Sequence

A total of 21 *Brucella* strains (15 in *B. melitensis* and six in *B. suis*) were selected for whole-genome draft sequencing, since these strains were isolated from recent years and represented the predominant species in this region. Genomic DNA of these strains were extracted with the SDS method (Lim et al., 2016). The harvested DNA was detected using agarose gel electrophoresis and quantified by Qubit^®^ 2.0 Fluorometer (Thermo Scientific). Sequencing libraries were generated using NEBNext^®^ Ultra^TM^ DNA Library Prep Kit for Illumina (NEB, United States) in accordance with the manufacturer’s recommendations, and index codes were added to attribute sequences to each sample. The draft genome of 21 *Brucella* strains was sequenced using Illumina NovaSeq PE150 at the Beijing Novogene Bioinformatics Technology, Co., Ltd. Illumina PCR adapter reads and low-quality reads from the paired end were filtered for quality control using readfq (version 10). All good-quality paired reads were assembled using the SOAP *de novo* (Li et al., 2008, 2010),^1^ SPAdes (Bankevich et al., 2012),^2^ and ABySS (Simpson et al., 2009)^3^ into a number of scaffolds. Filtered reads were then subjected to gap-closing. Draft genomic alignment between the sample genome and reference genome [*B. melitensis* 16M (Assembly ID: GCA_000007125.1) and *B. suis* 1330 (Assembly ID: GCA_000007505.1)] were performed using the MUMmer (Kurtz et al., 2004) and LASTZ (Chiaromonte et al., 2002) tools. Whole-genome SNPs were found using the results of genomic alignment among samples by the MUMmer and LASTZ, as mentioned above. In addition, 132 *B. melitensis* and 31 *B. suis* genomes were retrieved from GenBank and used for comparison and preliminary phylogenetic analyses. The phylogenetic tree was constructed using the TreeBeST (Vilella et al., 2009) based on Maximum-Likelihood Phylogenies (PHYML) with 1000 bootstrap replicates. Subsequently, the *B. melitensis* strains present discernible genetic differences in the SNP phylogenetic tree were removed; then, a simple phylogenetic tree was reconstructed based on 34 *B. melitensis* strains (Supplementary Table S5).

## Results

### Identification and Distribution of *Brucella* Strains

**FIGURE 1:**
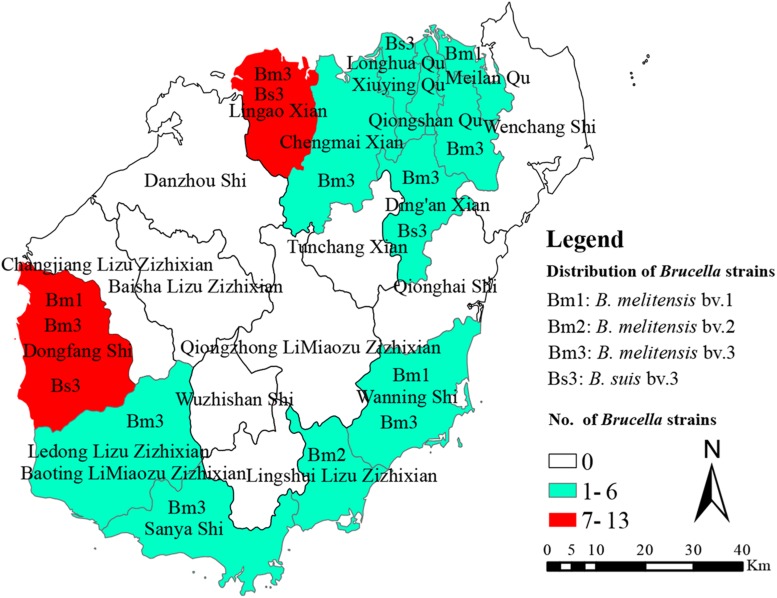
The geographic distribution of *Brucella* samples in Hainan, China. The map of Hainan province in this study was download from open source map (https://commons.wikimedia.org/wiki/Atlas_of_the_world).

All of the 41 *Brucella* strains were identified as *B. melitensis* bv. 1 (*n* = 5), *B. melitensis* bv. 2 (*n* = 1), *B. melitensis* bv. 3 (*n* = 23), and *B. suis* bv. 3 (*n* = 12) using classical biotyping methods (Table 1). From 2009 to 2019, a total of 41 *Brucella* strains were isolated. The number of strains isolated included 4 strains in 2009, 2 strains in 2010, 4 strains in 2012, 11 strains in 2013, 5 strains in 2014, 1 strain in 2015, 2 strains in 2016, 6 strains in 2017, 4 strains in 2018, and 2 strains in 2019. The 41 *Brucella* strains were observed in nine counties in Hainan Province, including Ledong (*n* = 1), Lingshui (*n* = 1), Wanning (*n* = 2), Chengmai (*n* = 2), Dingan (*n* = 3), Sanya (*n* = 3), LinGao (*n* = 7), Haikou (*n* = 8), and Dongfang (*n* = 13), and the original locations associated with the other strains are unknown (Figure 1).

**TABLE 1:** Biotyping characteristics of *Brucella* species isolates in Hainan Province, China.

**Strain no.**	**Growth characteristics**				**Monospecific sera**			**Phages lysis testing**			**Interpreted**
				
	**CO_2_ requested**	**H_2_S**	**BF**	**TH**	**A**	**M**	**R**	**Tb**	**BK_2_**	**Wb**	
BA	+	+	+	−	+	−	−	CL	CL	CL	*B. abortus 544*
BM	−	−	+	+	−	+	−	NL	CL	NL	*B. melitensis16M*
BS	−	+ +	−	+	+	−	−	NL	CL	CL	*B. suis 1330*
5	−	−	+	+	−	+	−	NL	CL	NL	*B. melitensis bv. 1*
1	−	−	+	+	+	−	−	NL	CL	NL	*B. melitensis bv. 2*
23	−	−	+	+	+	+	−	NL	CL	NL	*B. melitensis bv. 3*
12	−	−	+	+	+	−	−	NL	CL	CL	*B. suis bv. 3*

*Description of data: Strain No., the number conferred to isolates; BF, basic fuchsin at 20 μg/ml (1/50,000 w/v); TH, thionin at 20 μg/ml (1/50,000 w/v); Phages, Tb = Tbilisi, BK_2_ = Berkeley type 2, Wb = Weybridge; CL, confluent lysis; NL, no lysis; RTD, routine test dilution; +, positive (serum agglutination positive); −, negative (serum agglutination negative).*

### MLVA-16 Genotyping Results

**FIGURE 2:**
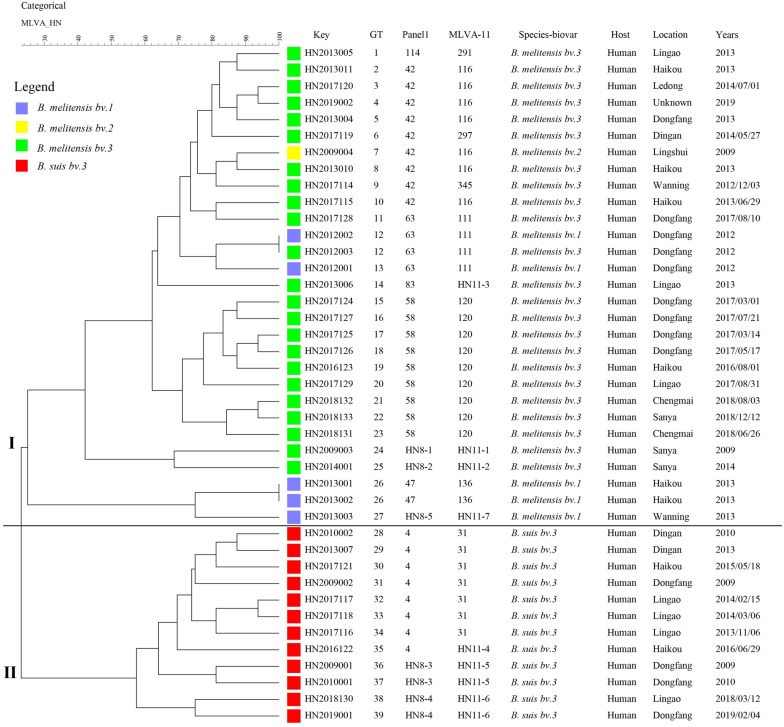
A dendrogram based on the MLVA-16 genotyping assay (UPGMA method) showing the relationships between the 41 *Brucella* isolates. The columns show the identification numbers (Key), MLVA-16 genotypes (GT), panel 1 genotypes and MLVA-11 (panels 1 and 2A) genotypes, species biovar, host, location, and the year the strains were isolated.

**FIGURE 3:**
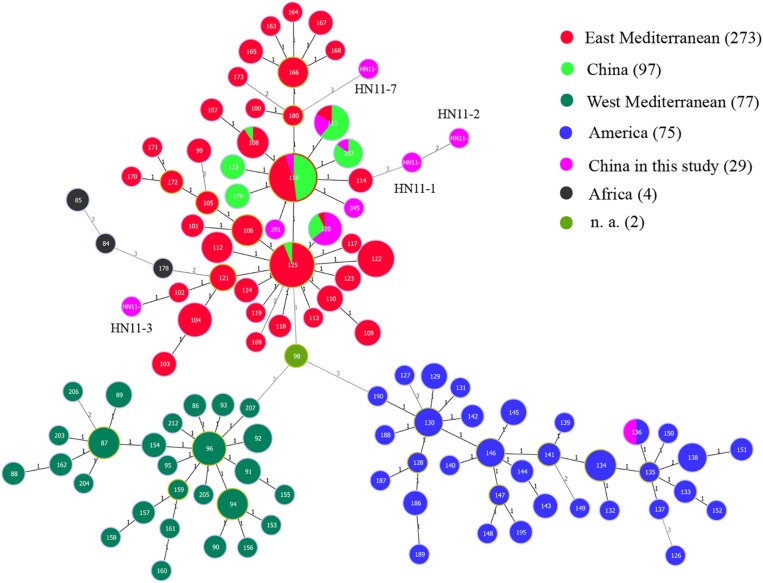
A MST for *B. melitensis* using the MLVA-11 data with the East Mediterranean group (red), the America group (blue), the Africa group (black), and the West Mediterranean group (blackish green) and compared to the China genotypes (green) and China isolates from our study (pink). n. a., not available (light green).

Based on the complete MLVA-16 scheme, 41 strains were divided into two groups (I and II). Twenty-nine *B. melitensis* strains clustered in group I, and all *B. suis* strains clustered in group II (Figure 2). A total of 29 *B. melitensis* isolates clustered into 27 distinct MLVA-16 genotypes (GT1-27), and 25 of them were represented by singular independent strains. The other two genotypes (GT12 and GT26) were shared by two isolates obtained from the same family (Figure 2). Following analysis of the data pertaining to MLVA-8, 29 *B. melitensis* strains formed nine MLVA-8 genotypes, including six known genotypes [83 (*n* = 1), 114 (*n* = 1), 47 (*n* = 2), 63 (*n* = 4), 58 (*n* = 9), and 42 (*n* = 9)] and three novel genotypes: HN8-1 (2-3-4-11-2-2-3-2; *n* = 1), HN8-2 (2-2-4-10-2-2-3-2, *n* = 1), HN8-5 (3-4-2-12-2-2-3-2, *n* = 1). The phylogeographic relationships of the 29 Hainan *Brucella* isolates were assessed with MLVA-11, and 11 different genotypes were identified (Figure 3). Seven of these genotypes were previously described (111, 116, 120, 136, 291, 297, and 345) and the remaining four novel (HN11-1 to HN11-3 and HN11-7) genotypes represent one to two strains with single locus variants of these genotypes. Genotypes 120 and 116 were the predominant genotypes representing 55% (16/29) of the total isolates; the two genotypes were distributed in six areas of Hainan Province. Minimum spanning tree analysis revealed that the MLVA-11 genotypes of *B. melitensis* strains were clustered into the “East Mediterranean” and “America” groups and strains from the former were dominant (Figure 3). In *B. melitensis* strains, the HGDI value of seven loci was >0.5, and the HGDI value of Panel 1, MLVA-11, and MLVA-16 was 0.8054, 0.8424, and 0.9951, respectively (Table 2).

**TABLE 2:** HGDI values of 29 *B. melitensis* isolates and 12 *B. suis* isolates.

**MLVA-16**	***B. melitensis***		***B. suis***	
	**Locus**	**HGDI ^*a*^**	**Locus**	**HGDI ^*a*^**
Panel 1	bruce06	0.3103	bruce06	0.3030
	bruce08	0.3128	bruce08	0.0000
	bruce11	0.3103	bruce11	0.0000
	bruce12	0.1995	bruce12	0.5455
	bruce42	0.5320	bruce42	0.0000
	bruce43	0.6158	bruce43	0.0000
	bruce45	0.0000	bruce45	0.0000
	bruce55	0.1330	bruce55	0.0000
Panel 2A	bruce18	0.1921	bruce18	0.0000
	bruce19	0.2537	bruce19	0.0000
	bruce21	0.1970	bruce21	0.1667
Panel 2B	bruce04	0.7906	bruce04	0.8030
	bruce07	0.6281	bruce07	0.7727
	bruce09	0.7340	bruce09	0.9242
	bruce16	0.8621	bruce16	0.8788
	bruce30	0.7734	bruce30	0.8485
MLVA	Panel1	0.8054	Panel1	0.5455
	MLVA-11	0.8424	MLVA-11	0.6515
	MLVA-16	0.9951	MLVA-16	1.0000

*^*a*^HGDI: Hunter–Gaston Diversity Index, which measures the variation of the number of repeats at each locus, and ranges from 0.0 (no diversity) to 1.0 (complete diversity).*

**FIGURE 4:**
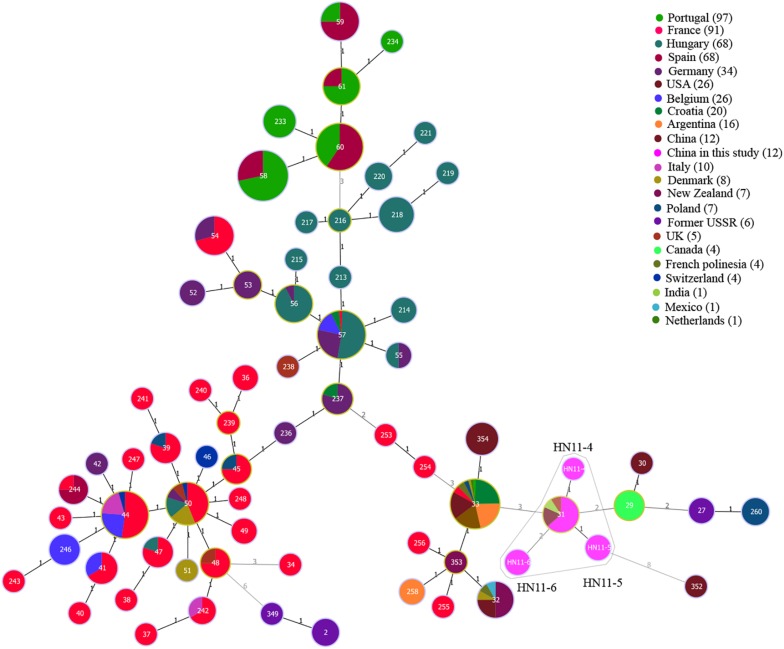
A MST for *B. suis* using the MLVA-11 data with the Portugal isolates (light green), France isolates (red), Hungary isolates (dark green), Spain isolates (Danish red), Germany isolates (dark purple), United States isolates (brown red), Belgium isolates (blue), Croatia isolates (emerald-green), Argentina isolates (orange), Italy isolates (dark pink), Denmark isolates (canary yellow), New Zealand isolates (dark Brown), Poland isolates (blue-gray), former USSR isolates (light purple), United Kingdom isolates (light red), Canada isolates (green), French Polynesia isolates (soil orange), Switzerland isolates (dark blue), India isolates (Yellow-green), Mexico isolates (lake blue), Netherlands isolates (green onion), China isolates (brown), and China isolates from our study (pink).

Among 12 *B. suis* strains, 12 single MLVA-16 genotypes (GT28-39) were observed (Figure 2). These strains were sorted into three MLVA-8 genotypes, 4 (*n* = 8), HN8-3 (2-3-4-12-3-1-5-2, *n* = 2), and HN8-4 (1-3-4-13-3-1-5-2, *n* = 2), and formed four MLVA-11 genotypes, one genotype was previously described (31) and the remaining three were novel (HN11-4 to HN11-6). MLVA-11 genotypes of *B. suis* belong to the genotype 31 clone group (Figure 4). In *B. suis* strains, all Panel 2B loci were > 0.77, 0.3030 in bruce06, 0.5455 in bruce12, and 0.1667 in bruce21. The other eight loci showed no diversity: the HGDI values of Panel 1, MLVA-11, and MLVA-16 were 0.5455, 0.6515, and 1.0000, respectively (Table 2). A dendrogram of the 41 *Brucella* strains shows the strain identification features, the MLVA-8 genotype, panel 1 + panel 2A genotype, their geographical origins, and the year of isolation (Figure 2).

### Relevance of Genotyping for Clinical Cases and Trace-Back Analysis of *Brucella*

GT4 represents single strains obtained from a patient’s blood and his household registry in Anhui Province. There was no contact history with the infected source, and he worked in Guangdong Province. This strain had completely identical MLVA-16 genotypes with strains 2011Jiang#013 and 2011Jiang#017 [MLVAbank (Brucella_4_3)] from Guangdong province. GT22 contained a single strain that was isolated from a sheep farm in Saya, and this strain belonged to MLVA-11 genotype 120, which was the dominant genotype in Hainan Province. There was a similar MLVA-16 genotype with strain 2011Jiang#059 [MLVAbank (Brucella_4_3)] from Yunnan Province, China. Two genotypes (GT32 and GT33) each represent single *B. suis* strains and were obtained from two patients in the same family. These people raised pigs, and two strains showed a single locus difference in highly variable bruce16 only.

### Epidemiological Relationships Between *Brucella* Strains

**FIGURE 5:**
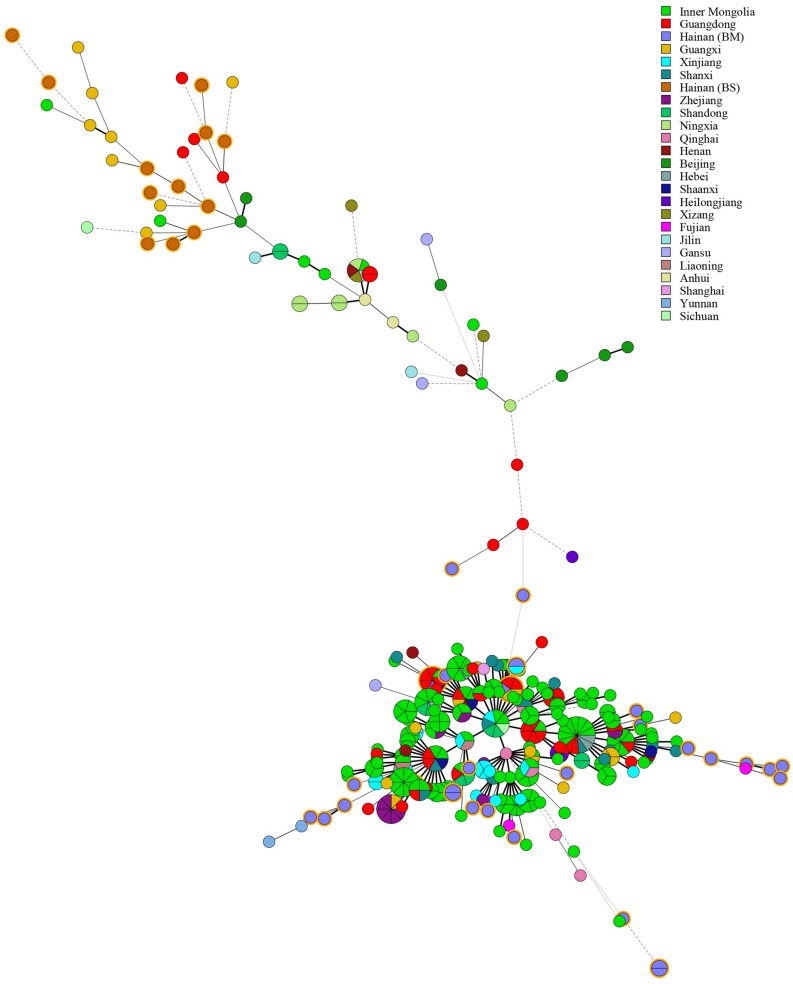
Minimum Spanning Tree characteristics based on *Brucella* strains throughout China. The tree was constructed using MLVA-16 data from 367 profiles of *Brucella* available in the MLVA international database. The nodes including isolates from this study are highlighted in yellow.

**FIGURE 6:**
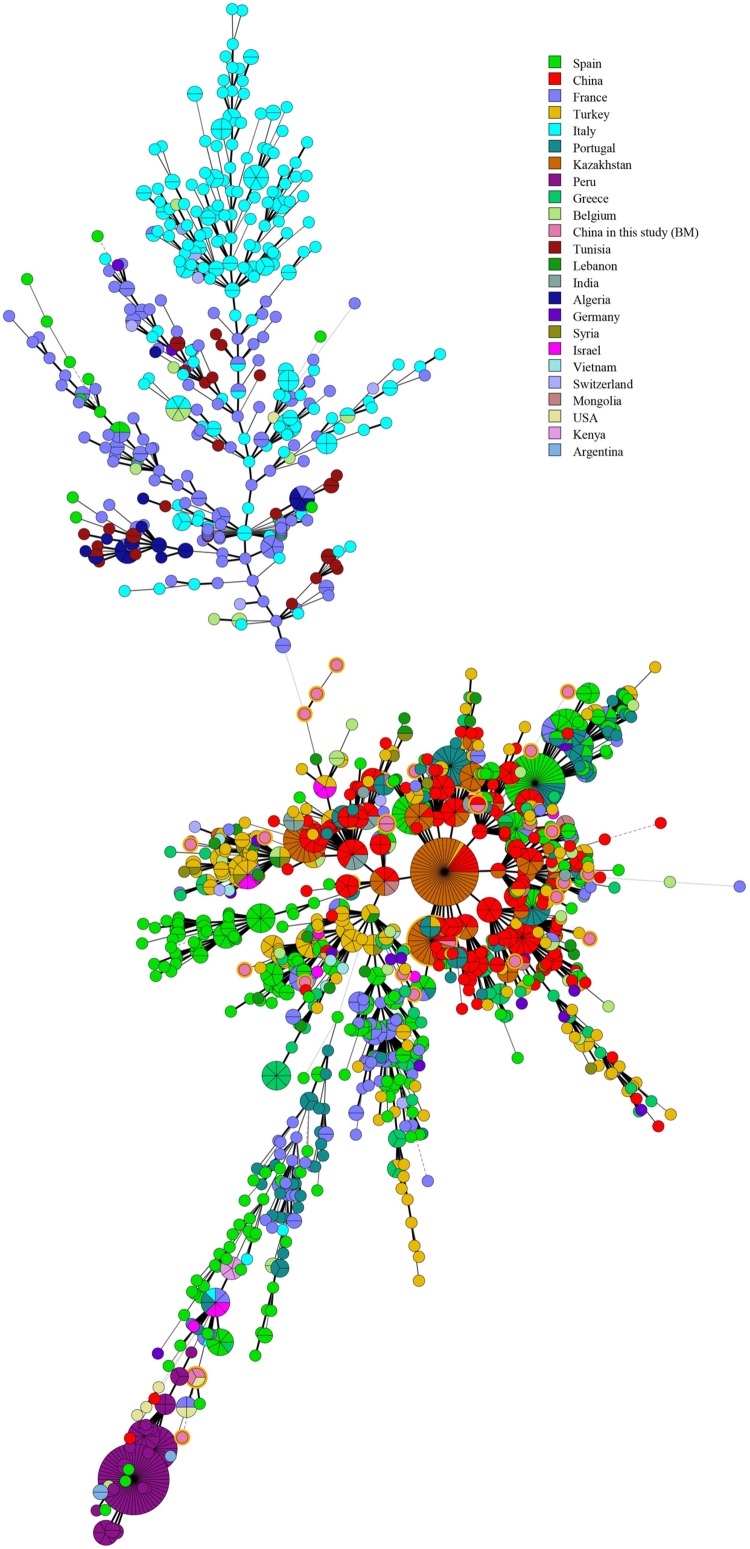
Minimum Spanning Tree characteristics based on global *B. melitensis* strains.

**FIGURE 7:**
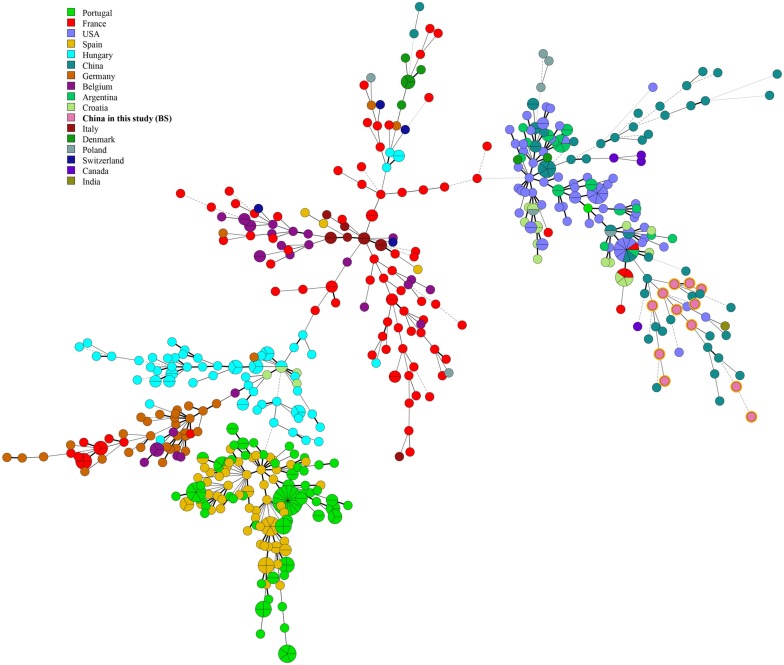
Minimum Spanning Tree characteristics based on global *B. suis* strains.

Multiple locus VNTR was used to determine the epidemiological links between 41 *Brucella* strains from Hainan Province and 367 *Brucella* strains (including *B. melitensis* and *B. suis*) from other provinces of China. Two shared genotypes were observed among the *B. melitensis* strains from this study and two different provinces, that is, Xinjiang and Guangdong (Figure 5). There is no shared MLVA-16 genotype among *B. suis* strains. Domestic isolates from Hainan Province were compared to foreign strains using an MLVA-16 assay. There were only two identical MLVA-16 genotypes from *B. melitensis* strains among those observed in this study and the strains from the United States and Argentina had discernible genetic difference from strains from Italy and France (Figure 6). However, *B. suis* in this study formed terminal subclades with strains from the United States and other parts of China, and it had significant genetic differences from strains from France, Italy, Spain, Germany, and Portugal (Figure 7).

### Phylogenetic Analysis Based on WGS-SNP

**FIGURE 8:**
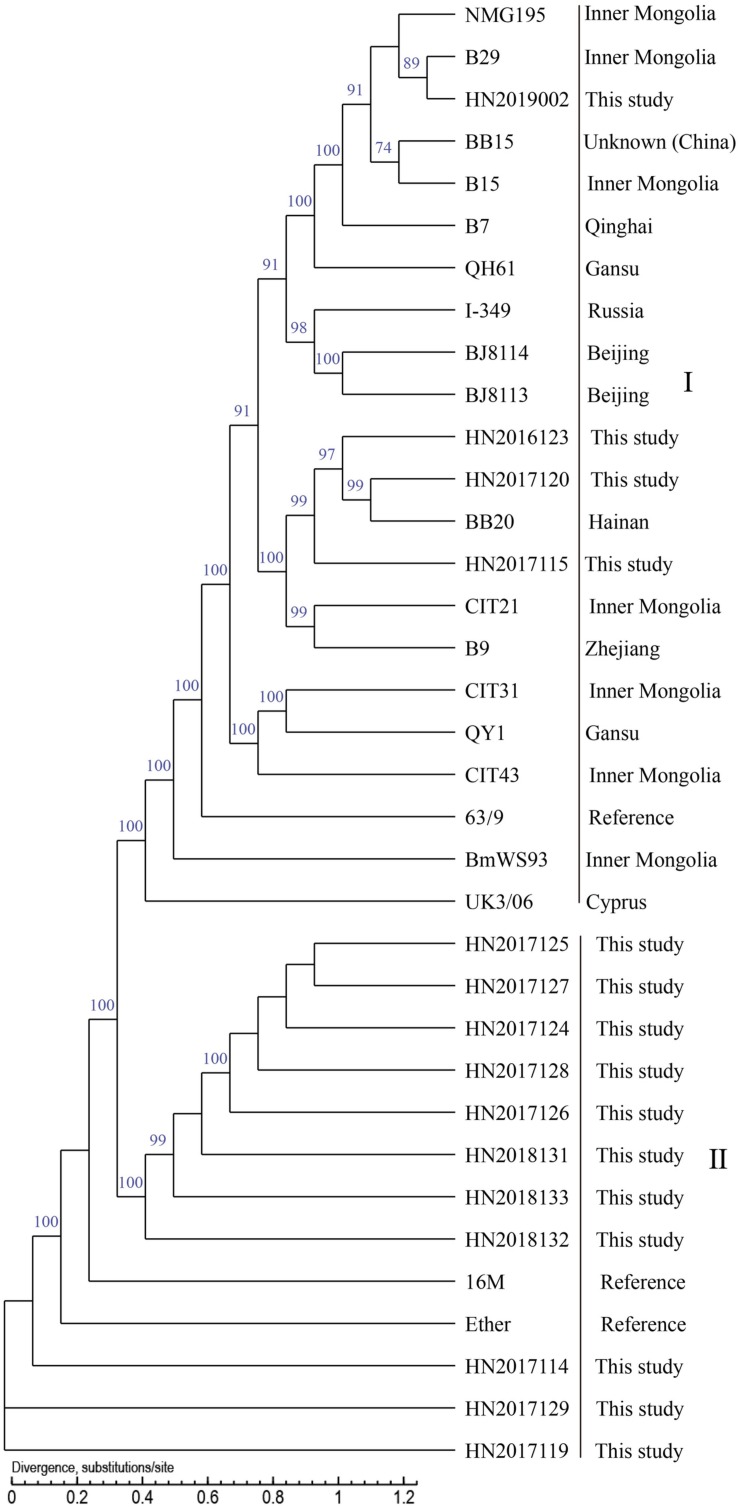
Phylogenetic trees of *Brucella melitensis* using SNPs from whole genomes.

**FIGURE 9:**
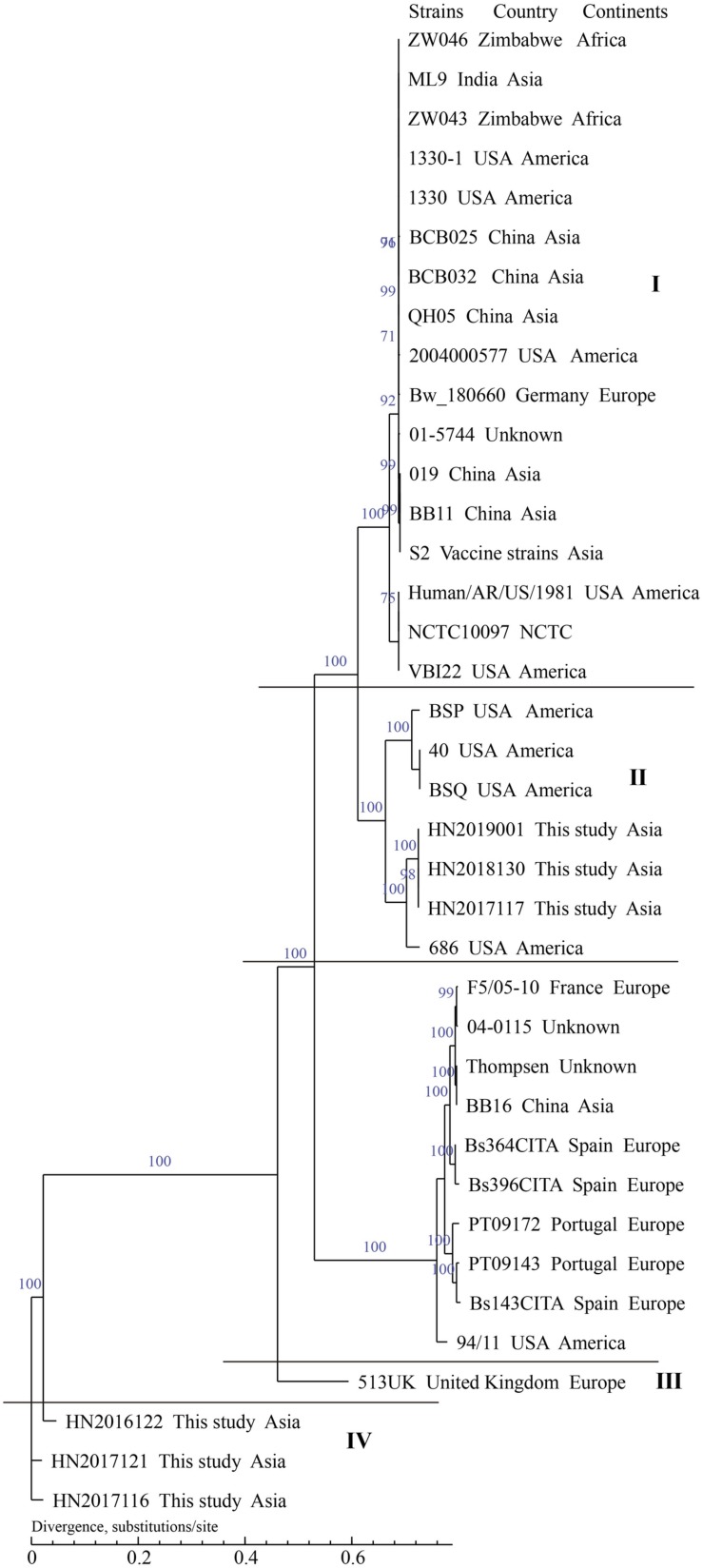
Phylogenetic trees of *Brucella suis* using SNPs from whole genomes. The dendrograms were generated using maximum likelihood with 1000 bootstrap replicates.

In the present study, the whole-genome draft sequences of 21 isolated strains, including 15 *B. melitensis* strains and 6 *B. suis* strains, were found. This was done to determine the genetic and evolutionary characteristics of this population. Briefly, the contigs from 21 strains ranged in length (>500 bp) from 19 to 25, with an average of 23. The average N50 Length (bp) (274,284–462,157) and N90 Length (bp) (102,688–117,359) were 329,690 (bp) and 116,049 (bp); the average GC content of 21 strains was 57.24% (57.18%–57.29%); the range of number of genes in these strains was 3210–3325, the average number of genes was 3276. *B. melitensis* 16M and *B. suis 1330* served as reference genomes for sequent datasets. The SNP numbers of 34 *B. melitensis* strains and 37 *B. suis* strains analyzed were 98,262 and 66,166. The whole-genome SNP analysis showed that 15 *B. melitensis* strains were grouped into two clusters (I and II) (Figure 8), 4 of which in cluster I were closely related with strains from Inner Mongolia, while the remaining 11 strains were divided into cluster II. However, the analyzed *B. suis* strains were sorted into four groups (I–IV) (Figure 9), strains from this study fell into groups II and IV, and three *B. suis* from group II were closely related with strains from the United States, while the other three strains in group IV showed an independent branch.

## Discussion

Brucellosis remains one of the major risks of public health in China, and human brucellosis has been reported in all 32 mainland provinces (regions) (Chen et al., 2014). Hainan province is located in the southernmost region of the nation and human brucellosis was rare in this region before 2009. The characterization of the geographic distribution and species of circulating strains is a critical process in understanding the epidemiology of brucellosis in the epidemic area (Kang et al., 2017). At least four species/biovars were detected in nine different areas, which suggests that *Brucella* strains were widely distributed in this province and exhibited high biovar diversity, which may have contributed to the spread and expansion of *Brucella* strains in this region. *B. melitensis bv. 3* (*n* = 23) and *B. suis bv. 3* (*n* = 12) were dominant species, and these results were different from studies from other major areas of China (Ma et al., 2016; Sun et al., 2016). Previous reports show that *B. melitensis* were the most common in the northwest and northeast China, but *B. suis* were often observed in the southern regions (Liu et al., 2018; Piao et al., 2018). This result indicates that brucellosis in Hainan has the unique epidemiology patterns of compatible southern and northern China. Comparative genomic analysis of the representative strains is needed to reveal the origin and phylogenetic profile. Moreover, the detection of brucellosis in animals is essential for the prevention of the disease. We consider that efficient preventive measures should be established to control the disease.

Pertaining to MLVA-8 data, 12 different MLVA-8 genotypes were identified, and 7 of them were previously described and widely distributed in other provinces in China. The remaining five genotypes were novel genotypes, which included three in *B. melitensis* and two in *B. suis*. Seven of 12 MLVA-8 genotypes were exclusively found in the Hainan Province, and these results suggest that the epidemiology of brucellosis in this region may be distinctive. Eleven MLVA-11 genotypes were found in strains from 29 *B. melitensis* isolates, suggesting that strains from this region have multiple geographic origins, but the East Mediterranean group was dominant. MLVA-11 genotype 136 was common in France, the United States, and Spain and belongs in the “America” group (Le Fleche et al., 2006). MLVA-11 genotypes 291, 297, and 345 were distributed in Guangdong, Shandong, Inner Mongolia, and other norther regions; MLVA-11 genotype 120 was predominantly distributed in Yunnan, Guangxi, Fujian, and Turkey. The strains representing this genotype may be imported from the vicinity of Yunnan, Guangxi, and Fujian provinces, which is consistent with the current epidemic features of human brucellosis. In addition, the HGDI value of seven loci in *B. melitensis* strains was over 0.5, and the HGDI value of Panel 1, MLVA-11, and MLVA-16 were all higher than those of strains from other regions within China (Jiang et al., 2011).

Four MLVA-11 genotypes were observed in 12 *B. suis* strains, of which the MLVA-11 genotype 31 was the dominant genotype. It was also common in the United States, United Kingdom, and India, indicating that some *B. suis* strains in this area had a common geographic origin with strains from these nations (Ferreira et al., 2017). Moreover, a total of seven new MLVA-11 genotypes were found in two species, four in *B. melitensis* strains and three in *B. suis* strains, and these genotypes may be unique in Hainan province. These data indicate that the *Brucella* strains in this study had multiple geographic origins, and this population exhibited characteristics of origin and evolution of co-existing imported and Hainan specific lineage. The diversity index of MLVA-16 in *B. suis* strains was 1.0000, which suggests that there was considerable genetic diversity among the *B. suis* population.

Based on the MLVA-16 scheme, 90% (25 + 12/41) strains formed unique genotypes and represented as single independent strains, which suggests that these strains had no related epidemiology and the epidemic characteristics of brucellosis in this province were predominated by sporadic occurrences (Liu et al., 2017). There were two shared genotypes (GT12 and GT26) that each consisted of two *B. melitensis* strains from the same family, and this finding confirmed the occurrence of the brucellosis outbreak events of the family. These two families were all engaged in breeding sheep and had regular contact with sheep every day, which is in agreement with information that was previously reported. Cluster outbreaks of a family of human brucellosis is common in southern China, and contact with infected sheep or its products is one of the main reasons for infection brucellosis (Kong, 2018). HN2019002 (GT4) was obtained from the blood of a patient in Hainan Provincial People’s Hospital and a household was registered to this patient in Anhui Province. During his work at Guangdong Province, the strain had a completely identical MLVA-16 genotype with strains from Guangdong Province, and we considered that this patient was infected with *Brucella* in Guangdong. HN2018133 (GT22) was isolated from the veterinary sample of a sheep farm in Sanya and showed almost identical genotypes with strains from Yunnan Province, China, and infected sheep imported from Yunnan Province was the source of infection for this case.

Among *B. suis* strains, both HN2017117 (GT32) and HN2017118 (GT33) strains were obtained from two patients in the same family, and there was only a single locus difference in the bruce16 locus, which suggests that there was a common source of infection among the two cases, but a mutation event occurs in highly variable loci (Kilic et al., 2011).

Based on an epidemiology investigation throughout China, two *B. melitensis* strains from this region had MLVA-16 genotypes identical to those of strains from Xinjiang and Guangdong Provinces, so these regions are the most likely sources of infection for human brucellosis in Hainan Province (Tian et al., 2017), but no identical MLVA-16 genotypes were found in *B. suis* species. Subsequent comparison of the relatedness of strains at a global level was performed, and two MLVA-16 genotypes from *B. melitensis* strains were observed between strains in this report and the United States and Argentina; *B. sui*s strains formed terminal subclades with strains from the United States and other provinces of China, indicating that strains from Hai province had visible genetic differences compared to strains from many parts of the globe.

Whole-genome SNP analysis showed that 15 *B. melitensis* strains were grouped into two clusters (I and II), which indicated that strains from each group had great genetic differences. The four strains in cluster I were closely related with strains from Inner Mongolia, which suggests that strains from Inner Mongolia were potential sources of infection for human brucellosis in Hainan Province. This result coincides with the conclusion from the MLVA analysis, where a previous study reported that strains from Inner Mongolia had a complete and identical MLVA-16 genotype with strains from many provinces in China (Liu et al., 2017); the other 11 strains were divided into cluster II, which occupied the basal node of the phylogenetic tree. We suggest that a comparison of the genome of *B. melitensis* strains from many different regions is warranted.

However, the analyzed *B. suis* strains were sorted into four groups (I–IV): *B. suis* from this study fell into two groups (II and IV) and three *B. suis* from group II were closely related to strains from the United States. Further investigation of the movement of animals, especially pigs, should be performed to identify the origins of these strains; the other strains in group IV showed an independent branch and are located in the basal node of the phylogenetic tree and had a previous historical occurrence. We observed that strains in this region exhibited unique characteristics of origin and evolution. Further efforts, examination of more strains, WGS, and collection of epidemiological data from the south are needed to accurately outline the pattern of transmission of brucellosis in Hainan, China.

Moreover, our study has some limitations. First, the data were collected from passive diagnosis that might have been influenced by laboratory tests or the physician’s understanding of the disease. Second, due to variability in the number of strains collected among different counties and for different years in this study, further research with additional strains is essential. Third, our research had no strains from animals in this region, and a genetic comparison in strains from a different host is lacking. Therefore, a whole-province survey on the human and animal infections with *Brucella* should be initiated.

## Conclusion

In the present study, molecular analysis of human *Brucella* strains from Hainan Province was performed. Our research showed that there was considerable diversity of biovar and genotypes among *Brucella* strains from Hainan Province. The epidemic characteristics of brucellosis in this region were predominated by sporadic traits, but also confirmed the occurrence of a brucellosis family outbreak. *B. melitensis* may have been imported from northern China. *B. suis* strains showed unique characteristics of origin and evolution, and they are of a lineage native to Hainan Province. Our work not only contributes to better understanding of the epidemiology of human brucellosis in Hainan Province, China, but it also provides considerable information that could be used to formulate control strategies for this disease.

## Data Availability Statement

The raw data supporting the conclusions of this article will be made available by the authors, without undue reservation, to any qualified researcher.

## Ethics Statement

The studies involving human participants were reviewed and approved by the Ethics Committees of the National Institute for Communicable Disease Control and Prevention. The patients/participants provided their written informed consent to participate in this study. Written informed consent was obtained from the individual(s) for the publication of any potentially identifiable images or data included in this article.

## Author Contributions

ZGL performed strain identification, MLVA genotyping and cluster analysis, and drafted the manuscript. XW and MW conducted epidemiological investigations and data analysis. XZ and HC prepared the DNA samples. ZJL and ZGL participated in the design of the study and critically reviewed the manuscript. ZJL and DL participated in the design of the study and managed the project. All authors have read and approved the final version of the manuscript.

## Conflict of Interest

The authors declare that the research was conducted in the absence of any commercial or financial relationships that could be construed as a potential conflict of interest.

## Footnotes

1 https://github.com/aquaskyline/SOAPdenovo2

2 http://cab.spbu.ru/software/spades/

3 http://www.bcgsc.ca/platform/bioinfo/software/abyss

## Abbreviations

HGDI: Hunter–Gaston discrimination index
MLVA: multiple locus variable-number tandem repeat analysis
MST: minimum spanning tree
PCR: polymerase chain reaction
SNPs: single-nucleotide polymorphisms
VNTR: variable-number tandem repeat analysis
WGS: whole-genome sequencing.
